# Cholesterol efflux capacity and its association with prevalent metabolic syndrome in a multi-ethnic population (Dallas Heart Study)

**DOI:** 10.1371/journal.pone.0257574

**Published:** 2021-09-21

**Authors:** Oludamilola Akinmolayemi, Suzanne Saldanha, Parag H. Joshi, Sneha Deodhar, Colby R. Ayers, Ian J. Neeland, Anand Rohatgi

**Affiliations:** 1 Department of Internal Medicine, Columbia University Irving Medical Center and NewYork-Presbyterian Hospital, New York, New York, United States of America; 2 Division of Cardiology, Department of Internal Medicine, University of Texas Southwestern Medical Center, Dallas, Texas, United States of America; 3 University Hospitals Harrington Heart and Vascular Institute and Case Western Reserve University School of Medicine, Cleveland, Ohio, United States of America; Medical University Innsbruck, AUSTRIA

## Abstract

Metabolic syndrome (MetS) is characterized by adiposity and atherogenic dyslipidemia consisting of elevated triglyceride and decreased high density lipoprotein cholesterol (HDL-C) levels however, cholesterol concentration alone does not reflect HDL functionality. Cholesterol efflux capacity (CEC) captures a key anti-atherosclerotic function of HDL; studies linking CEC to MetS have yielded inconsistent findings and lacked racial/ethnic diversity. The aim of this study was to evaluate the association between CEC and MetS in a large multi-ethnic population utilizing two different CEC assays interrogating overlapping but distinct reverse cholesterol transport pathways. A cross-sectional study was performed using the Dallas Heart Study cohort and cholesterol efflux was measured with radiolabeled and fluorescent cholesterol assays. The relationship between CEC and MetS was assessed using multivariable regression analyses. A total of 2241 participants were included (mean age was 50 years; 38% men and 53% Blacks). CEC was independently and inversely associated with MetS irrespective of efflux assay (CEC-radiolabeled, adjusted OR 0·71 [95% CI 0·65–0·80]. CEC-fluorescent, adjusted OR 0·85 [95% CI 0·77–0·94]). Both CEC measures were inversely associated with waist circumference and directly associated with HDL-C but not with other MetS components. There was an interaction by sex but not by race such that the inverse associations between CEC and MetS were somewhat attenuated in men (OR 0·86, 95%CI 0·74–1·01). In this large multi-ethnic cohort, impaired CEC is linked to MetS irrespective of efflux assay and race/ethnicity but less so among men. Future studies are needed to assess whether CEC mediates the atherosclerotic cardiovascular disease risk of MetS.

## Introduction

Metabolic syndrome (MetS) is a major risk factor for atherosclerotic cardiovascular disease (ASCVD) and type 2 diabetes [1–3]. Based on the 2007 to 2014 National Health and Nutrition Examination Survey (NHANES) data, the prevalence of MetS in the U.S. was approximately 34% however, the distribution was unequal among ethnic groups [4]. Insulin resistance is the hallmark phenotypic trait of MetS characterized by adiposity and atherogenic dyslipidemia consisting of elevated triglyceride and decreased high density lipoprotein cholesterol (HDL-C) levels [5–7] however, cholesterol concentration alone does not reflect HDL functionality.

Cholesterol efflux capacity (CEC) captures a key anti-atherogenic functionality as it characterizes the ability to promote reverse cholesterol transport–the movement of cholesterol from extra-hepatic cells in the periphery to the liver for excretion into the bile as either free cholesterol or bile acids—and assessed *in vitro* using both radiolabeled cholesterol efflux assay (CEC-radiolabeled) and fluorescent-labeled cholesterol efflux assay (CEC-fluorescent) [8,9]. CEC has been shown in several population-based studies to be inversely associated with and a predictor of ASCVD and cardiometabolic risk [10–17]. CEC is not reflected accurately by HDL-C or any other traditional lipid levels, which makes it important to understand how the function of HDL captured by CEC is linked to MetS.

Many studies assessing CEC in MetS have used an assay (CEC-radiolabeled) highly correlated with HDL-C, involved populations without ethnic diversity, and have been inconsistent in their results, with some finding decreased CEC and others finding increased CEC in those with MetS [18–24]. The aim of this study was to evaluate the association between CEC and MetS in a large multi-ethnic population utilizing two different CEC assays interrogating overlapping but distinct reverse cholesterol transport pathways.

## Materials and methods

### Study design and study population

The study design was a cross-sectional study of Dallas Heart Study phase 2 (DHS2) participants. Briefly, DHS2 is a longitudinal follow-up study of a subset of participants aged 35 to 70 who completed the Dallas Heart Study phase 1 (DHS1) and provided informed consent for a second comprehensive clinical study assessment with repeat data collection between September 2007 and December 2009. DHS1 is a multiethnic probability-based sample of Dallas County noninstitutionalized, English-speaking or Spanish-speaking adults aged 18 to 65 years enrolled between July 2000 and January 2002 that was weighted to include approximately 50% Blacks/African Americans. Recruitment procedures and study design have been reported previously [25]. The assessments performed during DHS2 included extensive health surveys, laboratory testing, and imaging studies during the visit of participants to the University of Texas Southwestern Medical Center. Data collection and analyses were performed at the time of the original study under the oversight of the Institutional Review Board (IRB) of the University of Texas Southwestern Medical Center and written informed consent was obtained from all the participants. Risk factor ascertainment and blood marker measurements other than cholesterol efflux measurements were performed at the time of the original study. All cholesterol efflux measurements were performed by our research team for this study and all data were fully anonymized before the authors of this study had access to the data therefore, IRB exempted this study from the need of ethics approval. For this study, participants with history of malignancies, End Stage Renal Disease (ESRD), Human Immunodeficiency Virus (HIV), and missing data for CEC were excluded yielding a final study sample size of 2241 participants with approximately 50% Blacks/African Americans, which of note is not representative of the larger U.S. population.

### Assessment of demographics, traditional cardiovascular risk factors, cardiometabolic variables, and lipids

Age, sex, race/ethnicity, post-menopausal status, anti-hypertensive medication use, statin use, smoking status, and drinking status were self-reported. Height and weight were measured using a standard physician’s scale and body mass index (BMI) was calculated as weight in kilograms divided by height in meters squared. Waist circumference was measured to the nearest centimeter at the level of the umbilicus. Physical activity was measured using Actical (Philips Respironics, Bend, Oregon) physical activity monitor that the participants wore on their wrist for 7 days and the monitors were set to record bodily movement, which was quantified as activity count (AC) per minute/day and moderate to vigorous activity (AC >1500 per minute) were recorded [26]. Blood pressures were obtained by an average of the third through fifth measurements of blood pressures. Fasting concentration of glucose (FBG), insulin, and glycated hemoglobin (HgbA1c) were determined from venous blood samples. Homeostatic model assessment of insulin resistance (HOMA-IR) was calculated using fasting insulin (μIU/ml) × fasting glucose (mmol/liter)/22·5. Plasma lipids were measured using standard enzymatic methods and have been described previously [25]. History of diabetes was defined by a fasting glucose level ≥126 mg/dL, non-fasting glucose of >200 mg/dL, HbA1c ≥ 6·5% or self-reported history of diabetes in addition to the use of any glucose lowering medication. History of cardiovascular disease (CVD) was defined as self-reported or adjudicated myocardial infarction, congestive heart failure (CHF)/CHF hospitalization, stroke, transient ischemic attack, peripheral revascularization, unstable angina, atrial fibrillation, coronary bypass graft (CABG) surgery, percutaneous coronary intervention, and other vascular events.

### Assessment of cholesterol efflux capacity and metabolic syndrome

Blood was collected into standard blood collection tubes containing ethylenediaminetetraacetic acid (EDTA) anticoagulant maintained at 4°C for less than 4 hours, then plasma was isolated and stored at -80°C for further analysis [25]. CEC was assessed *in vitro* using both CEC-radiolabeled assay and CEC-fluorescent assay by measuring the efflux of labeled cholesterol from mouse J774 macrophages (Table in S1 Table) to apolipoprotein B (ApoB)–depleted plasma from study participants (S1 Appendix). The mouse J774 macrophages were commercially available and purchased from ATCC® with catalog number TIB67, however the cell lines were not verified. The cholesterol efflux assays evaluate cholesterol efflux as mediated by multiple transporters and passive diffusion, although fluorescent-labeled cholesterol efflux assay has been shown to be more sensitive for ATP-binding cassette transporter A1 (ABCA1)-mediated efflux [24]. Individual efflux values were normalized to values obtained with a pool of 2% apoB-depleted plasma from selected controls thus making the efflux values unitless. The intra-plate coefficient of variability was 3.3% and inter-plate coefficient of variability was 7.4% for the CEC measurements. The details of both measurement methods have been described previously [24,27].

MetS, according to the Adult Treatment Panel (ATP) III criteria, was defined as having any 3 or more of the following criteria: waist circumference >102 cm in men or >89 cm in women; triglycerides ≥150 mg/dL; HDL-C <40 mg/dL in men or <50 mg/dL in women; blood pressure ≥130/≥85 mmHg; and fasting blood glucose (FBG) ≥100 mg/dL [28].

### Statistical analysis

CEC was analyzed as both continuous and categorical (based on quartiles) variables. MetS was analyzed as categorical variables based on presence/absence of MetS and increasing number of MetS components (MetS0 = participants without any MetS component; MetS1 = participants with one MetS component; MetS2 = participants with two MetS components; MetS3 = participants with three MetS components; and MetS4-5 = participants with four or five MetS components).

Demographic and clinical variables were compared across increasing quartiles of CEC using Jonckheere–Terpstra trend test. Categorical variables were presented as frequencies and percentages. Continuous variables were presented as means with standard deviations for normally distributed variables and medians with interquartile ranges for skewed variables. Multivariable logistic regression analyses were performed to assess the association between CEC (continuous and quartiles) and MetS status. Multivariable ordinal logistic regression analyses using a generalized link function were performed to assess the association between CEC (continuous) and increasing components of MetS.

Multivariable linear regression analyses were performed to assess the association between CEC (continuous) and the individual components of MetS (waist circumference, systolic blood pressure, diastolic blood pressure, triglycerides, HDL-C, and FBG). Regression models were adjusted for age, sex, ethnicity, physical activity, current smoking, current drinking, LDL-C, VLDL-C, post-menopausal status, and history of CVD. LDL-C and VLDL-C were included to account for total burden of atherogenic lipids without including lipids that are part of the outcome variable of MetS (HDL-C and triglyceride). Non-normally distributed continuous variables were log-transformed prior to use in regression analyses. Test for interaction was performed to identify effect modification of gender, ethnicity, history of CVD, and history of diabetes in the relationship between CEC and MetS. Stratified analysis was then performed to evaluate the association within the stratum of the interacting variables with significant interaction.

Standardized odds ratios (OR) with 95% confidence intervals (CI) were reported for the multivariable binomial and ordinal logistic regression models. Standardized regression coefficients (Std β) with 95% confidence intervals (CI) were also reported for linear regression models. The standardized measures of association corresponded to the impact of 1-SD increase in the independent variable on the variability of the dependent variable. Two-sided P values <0·05 was considered statistically significant. All statistical analyses were performed using SAS version 9·4 (SAS Institute Inc., Cary, North Carolina).

## Results

A total of 2241 participants were included in the study. The mean age was 50 years with 38% men and 53% Blacks. The mean values for CEC-radiolabeled and CEC-fluorescent for the participants were 0.93 and 0.85 respectively. The proportion of participants who met the criteria for MetS was 39%.

### Characteristics associated with Cholesterol Efflux Capacity (CEC) (Tables 1 and 2)

Low CEC-radiolabeled was associated with younger age, male sex, and Black race. In contrast, low CEC-fluorescent was associated with fewer Hispanic participants but not with Black or White race nor age or sex. Low CEC-radiolabeled and CEC-fluorescent were both associated with higher BMI, waist circumference, insulin level, and HOMA-IR. Low CEC measures were also linked to lower levels of all traditional lipids, including VLDL-C, and low percentage of current drinkers.

**Table 1:** Baseline characteristics of participants across increasing quartile of cholesterol efflux capacity–radiolabeled.

	Cholesterol Efflux Capacity–Radiolabeled				
Variables	Q1 (N = 553)	Q2 (N = 554)	Q3 (N = 554)	Q4 (N = 553)	ǂP Value for Trend (Two-sided)
**Cholesterol efflux capacity**	**0·72 ± 0·10**	**0·88 ± 0·03**	**0·99 ± 0·03**	**1·15 ± 0·10**	**<0·001**
Demographics/Traditional Cardiovascular Risk Factors					
**Age (year)**	**49 ± 11**	**49 ± 11**	**51 ± 11**	**51 ± 11**	**<0·001**
**Male (%)**	**44**	**37**	**40**	**33**	**0·001**
**Metabolic syndrome (%)**	**46**	**35**	**36**	**35**	**0·002**
**Post-menopausal (%)**	**27**	**33**	**35**	**38**	**0·001**
**Race/Ethnicity (%)**					
**Black**	**55**	**59**	**50**	**47**	**0·001**
**White**	**28**	**25**	**31**	**35**	**0·001**
Hispanic	14	14	17	16	0·314
History of cardiovascular disease (%)	10	6	8	10	0·919
History of diabetes (%)	16	15	18	18	0·202
Antihypertensive medication use (%)	39	34	35	38	0·999
Statin use (%)	18	16	18	18	0·821
Current smoker (%)	21	25	23	23	0·635
Systolic blood pressure (mmHg)	132 ± 20	133 ± 21	132 ± 19	134 ± 21	0·218
Diastolic blood pressure (mmHg)	81 ± 9	81 ± 9	81 ± 9	82 ± 10	0·575
Cardiometabolic Variables					
**Current drinker (%)**	**68**	**67**	**69**	**74**	**0·017**
Physical activity (activity count >1500 per min/day)*	28·3 (13·4, 52·3)	27·6 (13·8, 53·0)	27·7 (13·9, 51.0)	30·3 (14·6, 53·9)	0·324
**Body mass index (kg/m** ^**2**^ **)**	**32·4 ± 7·9**	**31·5 ± 7·5**	**30·7 ± 7·1**	**30·0 ± 7·0**	**<0·001**
**Waist circumference (cm)**	**99·6 ± 16·9**	**97·4 ± 17·1**	**96·6 ± 16·0**	**94·6 ± 16·0**	**<0·001**
Fasting blood glucose (mg/dL)*	94 (87, 102)	93 (86, 102)	94 (87, 105)	94 (88, 103)	0·106
**Insulin (uIU/mL)** *	**14·1 (8·9, 22·3)**	**12·5 (8·7, 18·5)**	**12·4 (8·1, 18·6)**	**11·8 (8·0, 17·9)**	**<0·001**
Hemoglobin A1c (%)	5·8 ± 1·1	5·7 ± 1·0	5·8 ± 1·2	5·8 ± 1·3	0·319
**Homeostasis model assessment of insulin resistance** *	**3·4 (2·0, 5·6)**	**2·9 (1·9, 4·6)**	**2·9 (1·9, 4·9)**	**2·8 (1·8, 4·8)**	**0·002**
Lipids					
**Total cholesterol (mg/dL)**	**179 ± 38**	**189 ± 36**	**197 ± 37**	**205 ± 40**	**<0·001**
**Triglyceride (mg/dL)** *	**96 (70, 133)**	**99 (71, 137)**	**106 (74, 149)**	**105 (76, 155)**	**<0·001**
**Very low-density lipoprotein cholesterol (mg/dL)** *	**19 (14, 27)**	**20 (14, 27)**	**21 (15, 30)**	**21 (15, 31)**	**<0·001**
**Low-density lipoprotein cholesterol (mg/dL)**	**110 ± 33**	**116 ± 34**	**118 ± 35**	**119 ± 38**	**<0·001**
**High-density lipoprotein cholesterol (mg/dL)**	**46 ± 12**	**51 ± 11**	**54 ± 13**	**60 ± 18**	**<0·001**

Data reported as mean ± SD, median (interquartile range), or percentage. Q1, first quartile; Q2, second quartile; Q3, third quartile; Q4, fourth quartile.

*Non-normally distributed variable.

ǂTest for intergroup differences performed using Jonckheere–Terpstra trend test.

Bolded variables represent significant associations.

**Table 2:** Baseline characteristics of participants across increasing quartiles of cholesterol efflux capacity–fluorescent.

	Cholesterol Efflux Capacity–Fluorescent				
Variables	Q1 (N = 560)	Q2 (N = 560)	Q3 (N = 561)	Q4 (N = 560)	ǂP Value for Trend (Two-sided)
**Cholesterol efflux capacity**	**0·56 ± 0·11**	**0·77 ± 0·05**	**0·92 ± 0·04**	**1·14 ± 0·15**	**<0·001**
Demographics/Traditional Cardiovascular Risk Factors					
Age (year)	50 ± 11	49 ± 11	49 ± 11	50 ± 11	0·795
Male (%)	40	36	38	40	0·747
Metabolic syndrome (%)	42	37	37	38	0·170
Post-menopausal (%)	35	32	34	32	0·570
Race/Ethnicity (%)					
Black	55	54	53	50	0·147
White	31	29	29	30	0·913
**Hispanic**	**12**	**15**	**15**	**18**	**0·015**
History of cardiovascular disease (%)	10	8	9	8	0·346
History of diabetes (%)	17	15	17	17	0·977
Antihypertensive medication use (%)	42	33	36	36	0·120
Statin use (%)	20	16	18	16	0·261
Current smoker (%)	23	23	22	24	0·968
Systolic blood pressure (mmHg)	133 ± 20	132 ± 21	133 ± 19	134 ± 21	0·172
Diastolic blood pressure (mmHg)	81 ± 10	81 ± 10	81 ± 9	81± 9	0·337
Cardiometabolic Variables					
**Current drinker (%)**	**67**	**66**	**73**	**72**	**0·032**
Physical activity (activity count >1500 per min/day)*	28·0 (14·0, 50·7)	28·2 (14·3, 51·5)	28·4 (14·3, 54·9)	28·5 (13·6, 53·7)	0·837
**Body mass index (kg/m** ^**2**^ **)**	**31·8 ± 7·4**	**31·4 ±7·7**	**31·2 ± 7·4**	**30·4 ± 7·3**	**<0·001**
**Waist circumference (cm)**	**98·9 ± 16·4**	**97·2 ± 16·7**	**96·9 ± 17·0**	**95·6 ± 16·3**	**0·001**
Fasting blood glucose (mg/dL)*	95 (88, 103)	93 (87, 101)	94 (87, 103)	94 (87, 103)	0·797
**Insulin (uIU/mL)** *	**13·1 (8·5, 19·9)**	**13·0 (8·7, 20·8)**	**12·9 (8·7, 20·7)**	**11·4 (7·9, 17·6)**	**0·005**
Hemoglobin A1c (%)	5·8 ± 1·1	5·8 ± 1·2	5·8 ± 1·1	5·8 ± 1·3	0·209
**Homeostasis model assessment of insulin resistance** *	**3·1 (1·9, 5·0)**	**3·1 (1·9, 5·2)**	**3·1 (1·9, 5·3)**	**2·8 (1·8, 4·5)**	**0·036**
Lipids					
**Total cholesterol (mg/dL)**	**185 ± 37**	**187 ± 36**	**195 ± 41**	**202 ± 39**	**<0·001**
**Triglyceride (mg/dL)** *	**97 (70, 132)**	**100 (72, 135)**	**100 (74, 143)**	**110 (79, 162)**	**<0·001**
**Very low-density lipoprotein cholesterol (mg/dL)** *	**19 (14, 26)**	**20 (14, 27)**	**20 (15, 28)**	**22 (16, 32)**	**<0·001**
**Low-density lipoprotein cholesterol (mg/dL)**	**112 ± 34**	**112 ± 34**	**118 ± 37**	**120 ± 37**	**<0·001**
**High-density lipoprotein cholesterol (mg/dL)**	**50 ± 14**	**52 ± 13**	**54 ± 16**	**55 ± 16**	**<0·001**

Data reported as mean ± SD, median (interquartile range), or percentage. Q1, first quartile; Q2, second quartile; Q3, third quartile; Q4, fourth quartile.

*Non-normally distributed variable.

ǂTest for intergroup differences performed using Jonckheere–Terpstra trend test.

Bolded variables represent significant associations.

### Cholesterol efflux capacity and prevalent MetS

**Fig 1:**
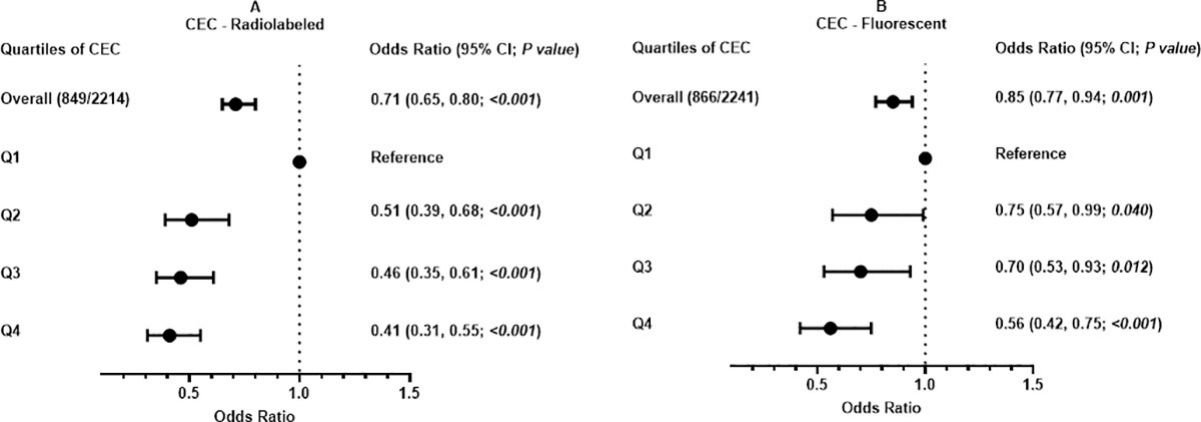
Association between cholesterol efflux capacity and metabolic syndrome. (A) CEC-Radiolabeled. (B) CEC-Fluorescent. Odds ratio = standardized odds ratio. Participant with no history of metabolic syndrome and Q1, participants in first quartile of cholesterol efflux capacity, as reference groups. Q2, second quartile; Q3, third quartile; and Q4, fourth quartile. Odds ratio adjusted for age, sex, ethnicity, physical activity, current smoking, current drinking, low-density lipoprotein cholesterol, very low-density lipoprotein cholesterol, post-menopausal status, and cardiovascular disease history.

Multivariable analysis revealed a significant inverse association between CEC-radiolabeled and MetS (OR per 1SD: 0·71; 95% CI, 0·65 to 0·80; *P*<0·001) (Fig 1A). Similar finding was observed between CEC-fluorescent and MetS (OR per 1SD: 0·85; 95% CI, 0·77 to 0·94; *P* = 0·001) (Fig 1B). When CEC was assessed as quartiles, there was a significant graded inverse association between increasing quartiles of CEC-radiolabeled and MetS (Fig 1A). Similar findings of an inverse graded association were observed for CEC-fluorescent and MetS (Fig 1B).

### Cholesterol efflux capacity and MetS components

**Fig 2:**
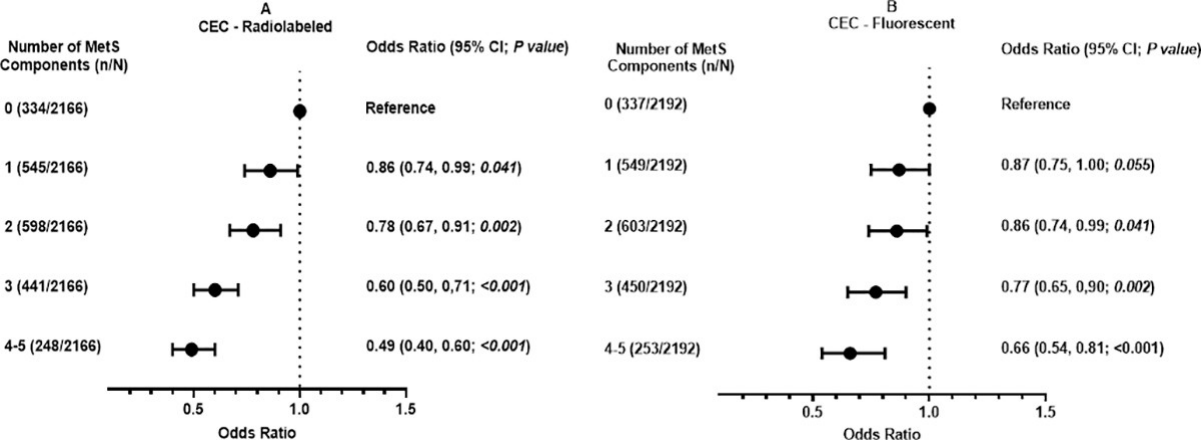
Association between cholesterol efflux capacity and increasing number of metabolic syndrome components. (A) CEC-Radiolabeled. (B) CEC-Fluorescent. MetS indicates metabolic syndrome. 0 = participants without any MetS component. 1 = participants with 1 MetS component. 2 = participants with 2 MetS component. 3 = participants with 3 MetS component. 4–5 = participants with 4 or 5 MetS component. (n/N) = total number of participants with MetS components/total number of participants. Odds ratio = standardized odds ratio. Odds ratio adjusted for age, sex, ethnicity, physical activity, current smoking, current drinking, low-density lipoprotein cholesterol, very low-density lipoprotein cholesterol, post-menopausal status, and cardiovascular disease history.

Among all participants, the magnitude of the association between both CEC-radiolabeled and CEC-fluorescent and MetS increased with increasing number of MetS components in adjusted analyses (from 1 component to 4–5 components; Fig 2). With respect to individual MetS components, both CEC measures were inversely associated with waist circumference and directly associated with HDL-C (Table 3). In the unadjusted model, CEC was associated with HDL-C (R = 0·36, *P*<0·001 for CEC-radiolabeled; R = 0·14, *P*<0·001 for CEC-fluorescent; Table in S2 Table). Neither CEC measure was associated with systolic blood pressure, diastolic blood pressure, triglyceride, or fasting blood glucose levels (Table 3).

**Table 3:** Association between cholesterol efflux capacity and individual components of metabolic syndrome.

	Cholesterol Efflux Capacity–Radiolabeled			Cholesterol Efflux Capacity–Fluorescent		
Variables	Std β	(95% CI)	P Value	Std β	(95% CI)	P Value
Diastolic blood pressure	0·01	-0·03 to 0·05	0·541	0·01	-0·03 to 0·05	0·670
Fasting blood glucose	0·04	-0·01 to 0·08	0·093	0·00	-0·04 to 0·05	0·850
**High-density lipoprotein cholesterol**	**0·38**	**0·34 to 0·41**	**<0·001**	**0·18**	**0·14 to 0·21**	**<0·001**
LogTriglyceride	-0·01	-0·03 to 0·01	0·207	0·00	-0·01 to 0·02	0·646
Systolic blood pressure	0·02	-0·02 to 0·05	0·445	0·02	-0·02 to 0·06	0·351
**Waist circumference**	**-0·12**	**-0·16 to -0·08**	**<0·001**	**-0·08**	**-0·12 to -0·04**	**<0·001**

Std β = standardized regression coefficient· β adjusted for age, sex, ethnicity, physical activity, current smoking, current drinking, low-density lipoprotein cholesterol, very low-density lipoprotein cholesterol, post-menopausal status, and cardiovascular disease history. Bolded variables represent significant associations.

### Assessment of effect modification on cholesterol efflux capacity and MetS

**Fig 3:**
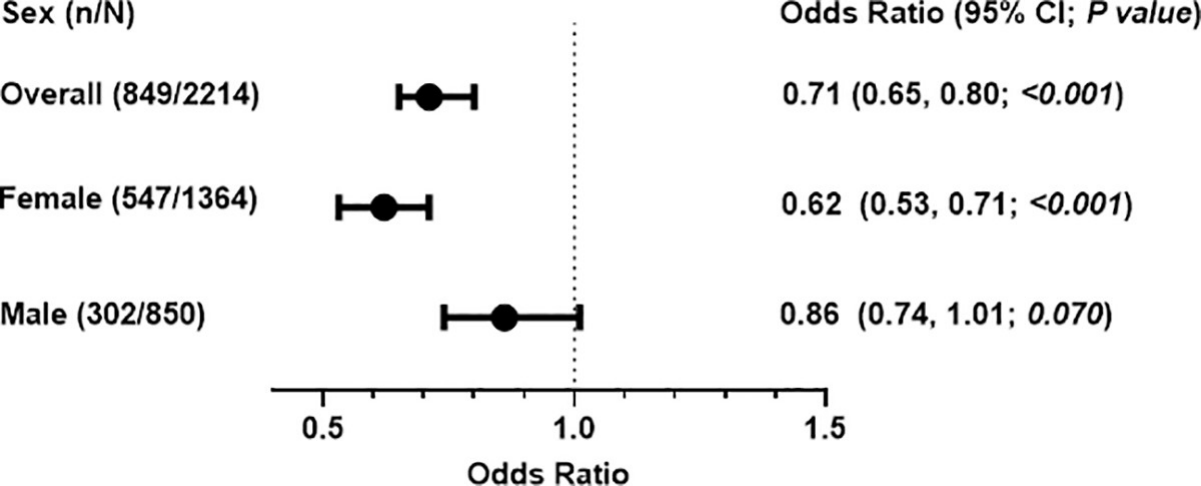
Association between cholesterol efflux capacity–radiolabeled and metabolic syndrome by gender. (n/N) = participants with metabolic syndrome/total number of participants within group. Odds ratio = standardized odds ratio. Odds ratio adjusted for age, sex, ethnicity, physical activity, current smoking, current drinking, LDL-C, VLDL-C, post-menopausal status, and history of CVD.

Finally, there was a significant interaction between CEC-radiolabeled and sex in its relationship with MetS (*P* for interaction = 0·04) with no significant interaction observed for CEC-fluorescent and sex (Table 4). In addition, there was no significant interaction between CEC and other risk factors such as LDL-C, VLDL-C, race/ethnicity, history of CVD, and history of DM (Table 4). In stratified analysis, the association between CEC-radiolabeled and MetS was preserved among females but was attenuated among males (For females, OR 0·62; 95%CI, 0·65 to 0·80. For males, OR 0·86; 95%CI, 0·74 to 1·01) (Fig 3).

**Table 4:** Test for interaction between cholesterol efflux capacity and other covariates in its association with metabolic syndrome.

	Cholesterol Efflux Capacity–Radiolabeled	Cholesterol Efflux Capacity–Fluorescent
Variables	P Valueǂ	P Valueǂ
**CEC x Male**	**0·04**	0·11
CEC x Very low-density lipoprotein cholesterol	0·15	0·11
CEC x Low-density lipoprotein cholesterol	0·16	0·10
CEC x History of diabetes	0·43	0·28
CEC x History of cardiovascular disease	0·60	0·33
CEC x Black	0·99	0·98

CEC, cholesterol efflux capacity.

ǂAdjusted for age, sex, ethnicity, physical activity, current smoking, current drinking, low-density lipoprotein cholesterol, very low-density lipoprotein cholesterol, post-menopausal status, and cardiovascular disease history.

P values are for the interaction terms listed within these multivariate models.

Bolded variables represent significant associations.

## Discussion

This study explored the relationship between cholesterol efflux capacity (CEC), measured by two distinct labeling methods, and prevalent metabolic syndrome (MetS) in a large, multi-ethnic cohort. CEC was found to be inversely associated with prevalent MetS, irrespective of efflux assay. In addition, there was a significant decrease in odds of having MetS components with increasing CEC. Furthermore, cholesterol efflux capacity was found to be directly associated with HDL-C and inversely with waist circumference but not with other components of MetS.

MetS is a composite of risk factors that include elevated fasting blood glucose, blood pressure, waist circumference, and triglyceride as well as low HDL-C. MetS is a strong risk factor for cardiovascular disease and type 2 diabetes. In several population-based studies involving participants who were free of cardiovascular disease and diabetes at baseline, MetS was found to be associated with increased risk of incident cardiovascular disease, morbidity and mortality, and prevalent atherosclerosis [1–3]. In a longitudinal study involving middle-aged adults without cardiovascular disease or type 2 diabetes at baseline, MetS was associated with an increased risk of type 2 diabetes irrespective of gender [1].

MetS represents an increased insulin resistance state and is defined by an atherogenic dyslipidemia consisting of low HDL-C levels, elevated triglyceride levels, and elevated small dense LDL particles. While low HDL-C is a strong risk marker for cardiometabolic disease, it does not adequately reflect efficiency of the reverse cholesterol transport pathway, which is purportedly anti-atherogenic. Movement of cholesterol from the periphery to the circulation is the key first step of this pathway, and ex vivo assays attempt to capture the efficiency of this first step in reverse cholesterol transport by quantifying the ability of plasma to accept cholesterol from standardized donor cells, termed in the literature as cholesterol efflux capacity (CEC). Several pathways have been shown to mediate cholesterol efflux, namely scavenger receptor class B type 1 (SR-B1), ATP binding cassette transporter G1 (ABCG1), ATP binding cassette transporter A1 (ABCA1), and aqueous diffusion [9,29,30]. Among these pathways, the ABCA1 pathway has been shown to play a major role in the maintenance of normal cholesterol levels in tissues. In mouse models with knocked out ABCA1 gene and among individuals with complete loss of ABCA1 function (Tangier disease), accumulation of large amounts of cholesterol in macrophages have been observed [31,32]. Most studies assessing CEC generally use radiolabeled cholesterol to measure cholesterol efflux, which reflects all cholesterol transport pathways to all sizes of apolipoprotein A-I containing particles and is moderately correlated with HDL-C levels (R ~ 0·4–0·5) [24]. The use of a fluorescent-labeled cholesterol shifts the focus toward pathways more specific for smaller, lipid-poor particles, resulting in much lower correlation with HDL-C levels in most studies and mild to moderate correlation with the radiolabeled assay [24]. Indeed, our study in a large, multi-ethic, population-based cohort with more heterogeneity revealed stronger associations between HDL-C and CEC-radiolabeled than with CEC-fluorescent and almost no association between the two CEC measures (Table in S2 Table). Lastly, most studies use plasma depleted of apolipoprotein B to focus on HDL particles as the main acceptors in plasma. Some studies use whole plasma, which contains more cholesterol acceptors and result in relationships that are not always consistent with those seen in studies using plasma depleted of apolipoprotein B [24]. Sankaranarayanan et al showed that apoB-containing lipoprotein may act as cholesterol acceptors. In a small sample of participants, they found the apoB-containing lipoproteins contributed about 25% of fluorescent-labeled cholesterol efflux and 42% of radiolabeled cholesterol efflux [24]. Our study served to address the discrepancy in these studies by studying both radiolabeled and fluorescent labeled cholesterol concurrently in the same study population. We chose to use plasma depleted of apolipoprotein B to focus the findings on acceptors less reflective of triglyceride-rich lipoprotein metabolism.

Results from studies assessing cholesterol efflux in MetS have been inconsistent, with some showing decreased and others increased CEC in individuals with MetS. Furthermore, most studies have utilized population samples without ethnic diversity. Gall et al included 1202 White patients with personal history of dyslipidemia and measured CEC from human THP-1 macrophages into whole serum using radiolabeled cholesterol. Among 307 individuals with MetS, CEC was lower, independent of age, LDL-C, lipid-lowering therapy, smoking status, and alcohol consumption [23]. Similarly, in a case-control study by Borja et al, HDL-apolipoprotein A-I exchange and ABCA1-specific CEC was measured in 74 White patients by radiolabeled efflux assay using transfected baby hamster kidney (BHK) cells overexpressing human ABCA1 and apoB-depleted serum. CEC was significantly reduced in 60 patients with MetS compared to the normolipidemic control subjects matched to the age and sex distributions in the MetS group [9]. Also, in a cross-sectional study by Annema et al involving a high cardiometabolic risk population of Caucasian origin enrolled in CODAM (Cohort on Diabetes and Atherosclerosis Maastricht), CEC was significantly lower in 297 subjects with MetS compared to subjects without MetS [20]. THP-1 macrophage foam cells and 2% apoB-depleted plasma were used in this study. Our study is consistent with the reported inverse associations between CEC and extends these findings in the following manner: 1) confirms this inverse relationship definitively in over 800 individuals with MetS; 2) establishes for the first time a lack of modification by race in a multi-ethnic population study; 3) reports an interaction by sex; and 4) reports consistent findings with two different cholesterol labels to determine CEC. Furthermore, our study reports for the first time a dose response with increasing number of MetS components, further providing evidence that low CEC is linked to not only presence of MetS but more severe MetS. Conversely, the findings from our study are inconsistent with the findings from the study by Dullaart et al that assessed the ability of whole plasma from metabolic syndrome subjects to promote CEC out of a cultured human fibroblast using the radiolabeled efflux assay. Their study involved a total of 170 White participants (76 with metabolic syndrome) and revealed preserved ability of plasma to promote CEC out of fibroblast among individuals with metabolic syndrome [22]. The findings from our study also are inconsistent with the findings from Alenezi et al involving 59 subjects (22 with metabolic syndrome) that showed preserved CEC using radiolabeled cholesterol efflux assay from fibroblasts to apo A-1 in serum of patients with metabolic syndrome [18]. It is important to note that these two studies used fibroblast cells as well as whole plasma/serum in determining CEC and also involved a small sample size. Since whole plasma contains VLDL and LDL, also cholesterol acceptors and receivers of cholesterol via CETP exchange from HDL, higher triglyceride levels promote exchange of cholesterol to these particles and enrichment of HDL with triglyceride. This triglyceride enrichment then leads to enhanced lipase action and hydrolysis of HDL particles, resulting in smaller ApoA-I containing particles which can participate in the ex vivo efflux assays to pick up cholesterol. The presence of elevated triglyceride can confound the measurement of CEC, and this may explain the findings in the study by Alenezi et al where the mean triglyceride level among patients with MetS was 340 mg/dL [22]. CEC has been shown to be preserved or increased in hypertriglyceridemic patients given preserved pre B-HDL levels and pre B-HDL formation [33]. Pre B-HDL serves as the initial acceptors of cholesterol from cells in the cellular efflux pathway and positively correlates with CEC [34]. Regarding the relationship between individual components of MetS and CEC, as expected, our study found a significant direct association between CEC and HDL-C while CEC was inversely associated with waist circumference adjusting for demographics, modifiable risk factors, lipids (other than HDL-C and Triglyceride), post-menopausal status, and CVD history. However, there was no significant association between efflux and other components. While the associations between HDL-C and efflux capacity are consistent with findings from other studies [20,23], our study precisely identifies that, adjusted for confounders, the relationship between impaired CEC and MetS is only driven by low HDL-C and increased waist circumference and not by the other components of the MetS. These findings suggest that lifestyle and pharmacologic interventions in those with MetS that most directly affect HDL-C and waist circumference may have the most impact on CEC.

It is worth mentioning that to the best of our knowledge, the impact of sex on the association between CEC-radiolabeled and MetS has not been reported elsewhere. However, some studies [20,23,35], including our study, have shown higher CEC-radiolabeled in females compared to males. The mechanism for this sex difference in CEC is still unclear, but Catalano et al showed an increased efflux capacity in females via SR-B1 pathway and males via ABCA1 pathway [36]. Therefore, future studies assessing the association between CEC and MetS should explore this sex effect.

There are several limitations of this study. First, this is a cross-sectional analysis of a large multi-ethnic cohort, and the intentional oversampling of Blacks do not reflect the general population, limiting the generalizability of this study. As expected in observation studies, temporality and causality cannot be properly assessed. Given the role of hormones like testosterone and soluble hormone binding globulin play in cholesterol efflux, it is important to note that their levels were not measured in this study. Lastly, the use of lipid lowering, glucose lowering, and anti-hypertensive medications were not adjusted for in the multivariable regression analyses, which could be potential confounders. It is worth mentioning that the sample size for this study is one of the largest with respect to MetS outcomes to further explore the association of CEC and presence and severity of MetS.

## Conclusion

This study demonstrated an inverse relationship between CEC and prevalent metabolic syndrome in a multi-ethnic population despite adjusting for demographics, modifiable risk factors, lipids not included in the MetS definition, post-menopausal status, and CVD history. CEC was also found to be positively correlated with HDL-C and negatively correlated with waist circumference. These findings were consistent regardless of the labeled cholesterol used in the efflux assay, which is a unique aspect of our study. Given inconsistencies in prior studies with fewer number of MetS participants, the findings from this study with a large number of MetS participants definitively confirm that impaired CEC is linked to MetS. Future studies are needed to assess whether CEC mediates the ASCVD risk of MetS.

## Supporting information

S1 Table Cultured cell line information. (PDF)

S2 Table Spearman correlation coefficients between CEC-radiolabeled, CEC-fluorescent, and HDL-C. (PDF)

S1 Appendix Cholesterol efflux capacity (CEC) measurements. (PDF)
