# Supplementary material for: Cholesterol efflux capacity and its association with prevalent metabolic syndrome in a multi-ethnic population (Dallas Heart Study)
Source: PLoS One. 2021 Sep 21;16(9):e0257574. doi: 10.1371/journal.pone.0257574 (PMC8454977; doi:10.1371/journal.pone.0257574)
Supplement: S1 Table — (PDF) [file pone.0257574.s001.pdf]

S1 Table. Cultured cell line information.

**Name and Catalogue #**	J774A.1 (ATCC® TIB67™)
**Cell Type**	Macrophages
**Vendor Source**	ATCC
**Organism**	*Mus musculus*, mouse
**Strain**	BALB/cN
**Tissue**	Ascites
**Disease**	Reticulum cell sarcoma
**Age/Gender**	Adult/Female
**URL**	https://www.atcc.org/products/all/TIB-67.aspx
**Cell line verification**	Cell line was not verified
