# Supplementary material for: Cholesterol efflux capacity and its association with prevalent metabolic syndrome in a multi-ethnic population (Dallas Heart Study)
Source: PLoS One. 2021 Sep 21;16(9):e0257574. doi: 10.1371/journal.pone.0257574 (PMC8454977; doi:10.1371/journal.pone.0257574)
Supplement: S2 Table — (PDF) [file pone.0257574.s002.pdf]

S2 Table. Spearman correlation coefficients between CEC-radiolabeled, CEC-fluorescent, and HDL-C.

	**CEC-radiolabeled**		**HDL-C**	
	**R**	**P Value**	**R**	**P Value**
**CEC-radiolabeled**	N/A	N/A	0.36	<0.001
**CEC-fluorescent**	0.10	<0.001	0.14	<0.001
