# Supplementary material for: Cholesterol efflux capacity and its association with prevalent metabolic syndrome in a multi-ethnic population (Dallas Heart Study)
Source: PLoS One. 2021 Sep 21;16(9):e0257574. doi: 10.1371/journal.pone.0257574 (PMC8454977; doi:10.1371/journal.pone.0257574)
Supplement: S1 Appendix — (PDF) [file pone.0257574.s003.pdf]

S1 Appendix. Cholesterol efflux capacity (CEC) measurements.

Fluorescent-labeled cholesterol efflux capacity (CEC) assay

Macrophage-specific CEC was measured using BODIPY-cholesterol [1]. J774 cells, derived from a mouse macrophage cell line, were initiated in Dulbecco's modified eagle medium containing 10% fetal bovine serum (FBS). When the cells were 90% confluent, they were plated into 96 well plates to a population of 7 x 10^6 cells per well and incubated overnight at 37ºC in the presence of 5% CO2. The following day, the cells were incubated for 1h with 0.025 mM BODIPY-tagged cholesterol (Avanti Polar Lipids), 0.2% bovine serum albumin (BSA), and 2µg/mL acetyl-Coenzyme A acetyltransferase (ACAT) Inhibitor (Sandoz, Sigma-Aldrich) in RPMI plus 1% FBS. Following this, the cells in each well were washed twice with MEM-HEPES buffer and incubated overnight at 37ºC in the presence of 5% CO2 in serum-free RPMI containing 0.3mM cAMP, 0.2% BSA, and 2µg/mL ACAT Inhibitor. Apolipoprotein B-depleted plasma from the study subjects was prepared using PEG precipitation [2]. The BODIPY-cholesterol cells were incubated with 2.8% apolipoprotein B-depleted plasma in MEM-HEPES buffer, 0.15mM cAMP and 2µg/mL ACAT Inhibitor for 4 hours at 37ºC. The resulting quantity of BODIPY-labeled cholesterol in the media was determined with a spectrophotometer and CEC was calculated as the amount of effluxed BODIPY cholesterol expressed as a fraction of the initial cell content of BODIPY cholesterol: [(millimoles of BODIPY-cholesterol in mediums containing 2.8% apolipoprotein B–depleted serum − millimoles of BODIPY-cholesterol in serum-free mediums) ÷ millimoles of BODIPY-cholesterol in cells extracted before the efflux step] × 100. Results were normalized to the measured efflux by a pooled reference apolipoprotein B-depleted plasma sample evaluated on every plate. All samples were run in duplicate and the average value was reported. The intra-plate coefficient of variability was 3.3% and inter-plate coefficient of variability was 7.4% for the CEC measurements.

Radiolabeled cholesterol efflux capacity (CEC) assay

Macrophage-specific CEC was measured using radiolabeled-cholesterol [1, 3]. J774 cells, derived from a mouse macrophage cell line, were initiated in Dulbecco's modified eagle medium containing 10% fetal bovine serum (FBS). When the cells were 90% confluent, they were plated into 96 well plates to a population of 7 x 10^6 cells per well and incubated overnight at 37ºC in the presence of 5% CO2. The following day, the cells were incubated overnight with 2 µCi of ^{3}H-tagged cholesterol (Perkin Elmer) and 2µg/mL ACAT Inhibitor (Sandoz, Sigma-Aldrich) in RPMI plus 1% FBS. Following this, the cells in each well were washed twice with MEM-HEPES buffer and incubated overnight at 37ºC in the presence of 5% CO2 in serum-free RPMI containing 0.3mM cAMP and 2µg/mL ACAT Inhibitor. Apolipoprotein B-depleted plasma from the study subjects was prepared using PEG precipitation [2]. The ^{3}H -cholesterol cells were incubated with 2.8% apolipoprotein B-depleted plasma in MEM-HEPES buffer, 0.15mM cAMP and 2µg/mL ACAT Inhibitor for 4 hours at 37ºC. The resulting quantity of ^{3}H-labeled cholesterol in the media was determined by liquid scintillation counting and CEC was calculated as the amount of effluxed ^{3}H cholesterol expressed as a fraction of the initial cell content of H3-cholesterol: [(microcuries of ^{3}H-cholesterol in mediums containing 2.8% apolipoprotein B–depleted serum − microcuries of ^{3}H-cholesterol in serum-free mediums) ÷ microcuries of ^{3}H-cholesterol in cells extracted before the efflux step] × 100. Results were normalized to the measured efflux by a pooled reference apolipoprotein B-depleted plasma sample evaluated on every plate. All samples were run in duplicate and the average value was reported. The intra-plate coefficient of variability was 3.3% and inter-plate coefficient of variability was 7.4% for the CEC measurements.

References

1. Sankaranarayanan S, Kellner-Weibel G, de la Llera-Moya M, Phillips MC, Asztalos BF, Bittman R, Rothblat GH. A sensitive assay for ABCA1-mediated cholesterol efflux using BODIPY-cholesterol. J Lipid Res. 2011 December 01;52(12):2332-40.

2. Asztalos BF, de la Llera-Moya M, Dallal GE, Horvath KV, Schaefer EJ, Rothblat GH. Differential effects of HDL subpopulations on cellular ABCA1- and SR-BI-mediated cholesterol efflux. J Lipid Res. 2005 October 01;46(10):2246-53.

3. Yancey PG, Kawashiri MA, Moore R, Glick JM, Williams DL, Connelly MA, Rader DJ, Rothblat GH. In vivo modulation of HDL phospholipid has opposing effects on SR-BI- and ABCA1-mediated cholesterol efflux. J Lipid Res. 2004 February 01;45(2):337-46.
